# Attitudes towards the administration of long-acting antipsychotics: a survey of physicians and nurses

**DOI:** 10.1186/1471-244X-13-58

**Published:** 2013-02-17

**Authors:** Paul Geerts, Guadalupe Martinez, Andreas Schreiner

**Affiliations:** 1 Janssen, Antwerpseweg 15-17, Beerse, 2340, Belgium; 2 Janssen, Paseo de las doce estrellas 5-7, Madrid, 28042, Spain; 3 Janssen, Johnson & Johnson Platz 5a, Neuss, D-41470, Germany

**Keywords:** Antipsychotic, Attitude, Long-acting injectable, Deltoid, Gluteal

## Abstract

**Background:**

Discontinuation of antipsychotic treatment for schizophrenia can interrupt improvement and exacerbate the illness. Reasons for discontinuing treatment are multifactorial and include adherence, efficacy and tolerability issues. Poor adherence may be addressed through non-pharmacological approaches as well as through pharmacological ones, ie ensured delivery of medication, such as that achieved with long-acting injectable (LAI) antipsychotics. However, attitudes of healthcare professionals (HCPs) towards LAI antipsychotics may influence their prescribing decisions and may influence medication choices offered to patients. We therefore conducted a survey to investigate factors driving LAI use as well as physician and nurse attitudes to LAI antipsychotics and to different injection sites.

**Methods:**

An independent market research agency conducted the survey of HCPs across Europe. Participants were recruited by telephone and completed the survey online. Using conjoint analyses (a multivariate statistical technique analysing preferences on the basis of ranking a limited number of attributes which are presented repetitively), attitudes to oral versus LAI medication and gluteal versus deltoid injection routes were assessed.

**Results:**

A total of 891 HCPs across Europe were surveyed. Of these, 40% would choose LAI antipsychotics for first episode patients whereas 90% would select LAI antipsychotics for chronic patients with two to five psychotic episodes. Dominant elements in antipsychotic choice were low sedation but no tardive dyskinesia, no or mild pain at injection and low risk of embarrassment or impact upon therapeutic alliance. Eighty-six per cent of respondents considered that having the choice of a deltoid as well as gluteal administration site was beneficial over not having that choice. Two thirds of respondents said they agreed that medication administration via the deltoid muscle may reduce social embarrassment associated with LAI antipsychotics and most respondents (61%) believed that administration of LAI antipsychotics into the deltoid muscle as opposed to the gluteal muscle may be more respectful to the patient.

**Conclusions:**

In this survey of physicians and nurses, attitudes towards LAI antipsychotics compared with oral medication were generally positive. Respondents considered that the availability of a deltoid administration route would offer increased choice in LAI antipsychotic administration and may be perceived as more respectful and less socially embarrassing.

## Background

Treatment continuation is a pervasive challenge in schizophrenia management [1]. Factors leading to treatment discontinuation include inability to adhere to medication regimens, lack of efficacy and tolerability issues [2-4]. Treatment discontinuation can lead to increased relapse rates [5,6] and poorer long-term outcomes [6]. With each successive relapse, chronicity is increased and response to treatment is reduced [7]. Ensuring treatment continuation through improved adherence is therefore a major objective in the treatment of schizophrenia and can impact on morbidity [6,8-11], attempted suicide [6,12], severity of violent behaviour [13], and patient satisfaction [9] as well as direct and indirect healthcare costs [14-16].

Long-acting injectable (LAI) antipsychotics are amongst the pharmacological options available to address adherence problems in patients. LAI antipsychotics offer a number of benefits compared with oral medication, including transparency of adherence [17], allowing healthcare professionals (HCPs) to be alerted and to intervene appropriately if patients fail to take their medication [18]. Other benefits of LAI antipsychotics include a reliable drug delivery with reduced peak–trough plasma levels [19], improved patient outcomes [20], improved patient and physician satisfaction [21] and lower relapse rates [22-24] than oral therapy. Furthermore, with LAI antipsychotics, there is regular contact between the patient and treatment team [25].

Several first-generation LAI antipsychotics and three second-generation LAI antipsychotics are currently available for administration into the gluteal muscle. Risperidone long-acting injectable (RLAI) is an aqueous-based, second-generation antipsychotic, available worldwide for both gluteal and deltoid injection [26]. The deltoid administration of RLAI has been shown to be bioequivalent to the gluteal administration [27]. Olanzapine pamoate, a prolonged-release suspension of olanzapine, is also available for gluteal injection [28]. A third atypical long-acting antipsychotic, paliperidone palmitate is available in the USA and Europe for injection in either the gluteal or deltoid muscle [29,30]. These new formulations provide patients and physicians with a choice of administration site for LAI antipsychotics.

Despite the adherence benefits of LAI antipsychotics, there is still considerable resistance to their first-line usage [31-33]. In one study directly comparing attitudes of psychiatrists, patients and relatives, patients were more negative towards long-acting or depot formulations than psychiatrists and relatives [32]. Experience of injectable antipsychotics can positively influence attitudes towards treatment, with up to 47% of patients experienced with this type of formulation favouring LAI antipsychotics over oral medication [33-35]. However, patients are often not informed of long-acting or depot formulations by their psychiatrist [32] and the attitudes of HCPs towards LAI medication may limit treatment choices offered to patients [36]. Educating staff, patients and families can help address prejudice and stigma towards LAI antipsychotics, increase familiarity and ultimately increase preference for this type of medication [37].

At the time of our study, studies investigating influencing factors and perceived preferences for antipsychotics were lacking. Attributes that may influence prescribing of long-acting formulations and patient acceptance include stigma [32], pain associated with injection [19] and embarrassment arising from the need to remove clothing for gluteal injections [25]. Although first-generation depot antipsychotics that use oil-based formulations are associated with pain on injection [19], aqueous-based formulations of LAI antipsychotics generally have good injection site tolerability [19]. Furthermore, injection into the deltoid muscle requires minimal removal of clothing [38]. A study reporting the findings from a Medication Preference Questionnaire for Patients cited the most common reasons for preferring the deltoid site (expressed by >25% of patients) as easier, less embarrassing, faster, or less painful than injection in the gluteal site [39]. The availability of both deltoid and gluteal formulations of LAI medication could therefore facilitate patient acceptance and long-term adherence to injectable antipsychotic medication. The objective of this present survey was to understand factors driving LAI use, as well as attitudes and preferences for different administration sites, both in comparison with oral medication and with other LAI medication. The survey used conjoint analysis, consisting of a series of trade-off questions. Participants were asked to choose between ‘bundles’ of defined attributes. This method permits the determination of dominant attribute levels from repetitive choice preferences [40].

## Methods

### Participants

The purpose of the present study was to understand physician and nurse attitudes and preferences for different administration sites of antipsychotic medication. An independent market research agency, funded by Janssen, conducted a survey of HCPs, including both physicians and nurses from across Europe, between November 2007 and January 2008. Participants were selected on two criteria: activity in the relevant clinical field, ie treating patients with schizophrenia, and willingness to participate in the study. They were recruited by telephone and asked to complete an online, choice-based survey to determine the relative importance of oral versus long-acting attributes on attitudes to antipsychotic use. The surveys were completed anonymously and responses were based on physician and nurse perceptions. Individual patient information was not collected and Institutional Review Board (IRB) review was not required. Participants were offered a small financial incentive of 50–70 Euros, depending on their country of origin, which they could nominate to donate to charity. Completed surveys were returned directly and blinded to the independent agency for data analysis.

### Data collection

The survey used choice-based conjoint analysis to gain insight into physician and nurse attitudes to the administration of long-acting antipsychotics. Conjoint analysis originated in mathematical psychology and was first introduced in marketing research. Nowadays, it is widely used in healthcare for eliciting patients’ and the community's preferences in the delivery of health services; establishing consultants’ preferences in priority setting; developing outcome measures; determining optimal treatments for patients; evaluating alternatives within randomized controlled trials; and establishing patients’ preferences in the doctor–patient relationship [40].

### Antipsychotic treatment characteristics

The characteristics of the antipsychotic treatment options included in this study are indicated below. Characteristics were chosen to elicit preferences for oral versus LAI antipsychotic medication (see subsection Choice-based conjoint exercise 1 - oral versus long-acting decisions) and deltoid versus gluteal administration (see subsection Choice-based conjoint exercise 2 - long-acting prescription decisions). Characteristics were derived from results previously published in the literature including findings from Medication Preference Questionnaires [16,22,29,32] and expert opinion obtained in advisory board settings.

#### Choice-based conjoint exercise 1 – oral versus long-acting decisions

Oral (is fixed)

1. Formulation

■ oral

2. Frequency of administration

■ once a day

3. Level of fluctuation of active compound in the blood plasma

■ high

4. Frequency of healthcare staff contact with patient

■ depends on level of care

5. Degree of certainty of assessing adherence with medication

■ low

Long-acting (varies)

1. Formulation

■ buttock muscle injection (gluteal)

■ deltoid muscle injection

2. Frequency of administration

■ once every 2 weeks

■ once every 3 weeks

■ once every 4 weeks

3. Level of fluctuation of active compound in the blood plasma

■ medium

■ low

4. Frequency of healthcare staff contact with patient

■ fixed: once per 2 weeks

■ fixed: once per 3 weeks

■ fixed: once per 4 weeks

5. Degree of certainty of assessing adherence with medication

■ high

#### Choice-based conjoint exercise 2 – long-acting prescription decisions

1. Routes of administration

■ gluteal muscle injection (buttock)

■ deltoid muscle injection

2. Dose scheme

■ once every 2 weeks

■ once every 3 weeks

■ once every 4 weeks

3. A significant degree of side effects

■ weight gain of more than 7% body mass

■ sedation

■ metabolic side effects (diabetes, hypercholesterolaemia, dyslipidaemia)

■ sexual side effects (prolactin)

■ extrapyramidal side effects

■ tardive dyskinesia

4. Pain at injection site

■ absent

■ mild

■ moderate

■ severe

5. Storage & transport

■ cold chain

■ no cold chain storage

6. Onset of action

■ within days

■ within weeks

7. Method of administration

■ injection technique with high risk of embarrassment & potential harm to therapeutic relationship

■ injection technique with low risk of embarrassment & potential harm to therapeutic relationship

8. Needle length

■ 2 ½ cm

■ 4 cm

■ 5 cm

9. Needle diameter

■ 19 G

■ 20 G

■ 21 G

### Assumptions underlying conjoint analysis

Conjoint analysis requires respondents to rate or rank a limited number of attributes related to antipsychotic features and make repetitive trade-offs between these attributes in order to reveal the relative importance of aspects of antipsychotic treatment [35]. The analysis assumes that each treatment option can be broken down into specific characteristics, and that each characteristic is defined by a number of levels. Levels refer to the range of plausible estimates for each characteristic. For example, the levels for the characteristic ‘pain at injection site’ might be absent, mild, moderate and severe depending on the specific treatment. The second assumption is that respondents have unique utilities for each attribute level. In this context, ‘utility’ is a number that represents the respondent’s preference for a particular characteristic, with higher utilities indicating greater preference and features most likely to drive treatment decisions. The utility scores represent the relative preference respondents have for the different levels within that specific attribute and can therefore only be compared within that attribute. For example, one can compare the scores of absent and mild pain within ‘pain at injection site’, but not between ‘mild pain at injection site’ and ‘side effect sedation’ as these are levels within different attributes. Within an attribute (eg pain at injection site), the least preferred level (severe pain) is always zero for the ease of understanding. The final assumption is that utilities can be combined across attributes. For example, if the sum of a patient’s utilities for the attributes of treatment A is greater than the sum of utilities for the attributes of treatment B, the patient should prefer treatment A to treatment B.

### Conjoint analysis questionnaire

**Figure 1:**
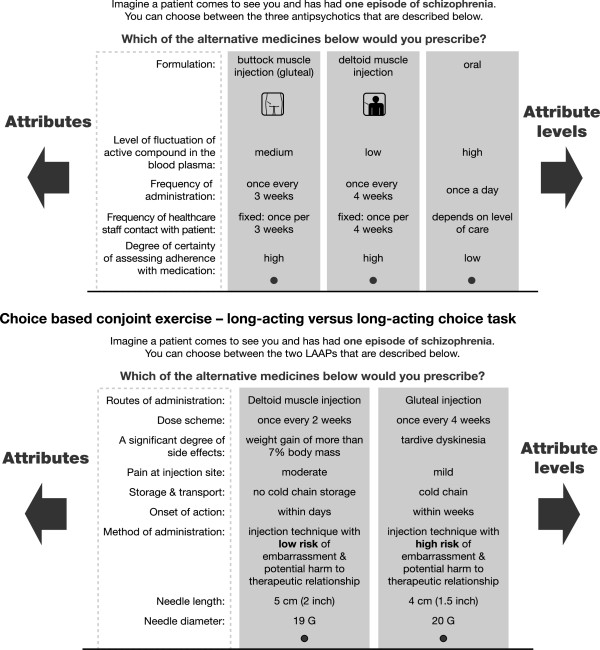
Choice-based conjoint exercise – oral versus long-acting choice task.

The choice-based conjoint task consisted of two groups of questions. Firstly, HCPs rated attributes relating to oral versus long-acting medications and secondly rated one long-acting medication versus another long-acting medication with varying attributes, such as dose interval, administration site and pain at injection site. Scenarios that described all possible configurations given the attributes and levels were included; an example of such a scenario is shown in Figure 1. The experimental design was optimized in such a way that all combinations of attribute levels were shown an approximately equal number of times to all respondents and, as far as possible, also, within a respondent, an equal number of times.

Respondents were also asked four questions regarding their own and their patients’ preferences and motivations. Additionally, respondents were asked to indicate, on a scale ranging from one to seven, whether they agreed or disagreed (1 = strongly disagree, 7 = strongly agree) with a series of statements regarding oral versus LAI medications, and one LAI medication versus another. Scores of 5, 6 and 7 were combined together to give an overall definition of ‘agree’ where 5 was ‘somewhat agree’, 6 ‘agree’ and 7 ‘strongly agree’.

### Analyses

Analyses were divided broadly into attitudes regarding oral versus long-acting medication, and regarding one long-acting medication versus another long-acting medication with varying attributes, such as dose interval or administration site. A logistic regression was used to analyse responses.

## Results

### Participants

One thousand seven hundred HCPs were approached to participate in this study; a total of 891 HCPs responded and completed the survey (a 52% response rate). The survey sample included physicians/specialists (78%) and nurses (22%). All physicians were psychiatrists or neuropsychiatrists. Respondents were from The Netherlands (n = 109), Belgium (n = 97), Germany (n = 182), Italy (n = 187), the UK (n = 83), France (n = 132), and the Nordic countries (n = 101). Among the participating HCPs, 75% had at least 8 years’ experience in psychiatry; 40% were based in a psychiatric hospital, 19% worked in a psychiatric department of a general hospital, 11% in private practice, 4% in a medical psychological centre, and 26% in another environment.

### Conjoint analysis

#### Oral versus LAI medication attributes

Antipsychotic attributes, levels and utility scores are listed in Table 1. The utility scores indicate the relative preference respondents have for different levels within an attribute. When compared to oral formulations, respondents had a greater preference for gluteal over deltoid formulation as shown by the utility score of +73.2; this represents the extra value that doctors and nurses ascribe to gluteal over deltoid formulation. A low fluctuation of active compound in the plasma and the frequency of regular contact with the patient were also identified as important attributes for antipsychotic treatment.

**Table 1:** Characteristics of antipsychotic treatment (oral versus LAI attributes)

**Attribute**	**Level**	**Utility scores**
Formulation	Buttock muscle injection (gluteal)	+73.2
	Deltoid muscle injection	0
Level of fluctuation of active compound in the blood plasma	Low	0
	Medium	+43.4

#### LAI versus LAI attributes

When considering LAI antipsychotic use, choice utilities revealed that side effects, pain at administration site, embarrassment and site of administration (gluteal versus deltoid) were all important factors (Table 2). Tardive dyskinesia was ranked as the most undesirable side effect, followed by metabolic issues (+42.3), extrapyramidal symptoms (+79.1), weight gain (+102.6), sexual side effects (+107.2) and sedation (+200.6). When considering pain at injection site, there was little difference in preference for no or mild pain at injection site (+158.8 versus +158.7). Preference data predicted that no/mild administration site pain, minimal risk of embarrassment/damage to the therapeutic relationship and some sedation but no other side effects were features of an ideal LAI.

**Table 2:** Characteristics of antipsychotic treatment (LAI versus LAI attributes)

**Attribute**	**Level**	**Utility scores**
Frequency of administration and healthcare staff contact with patient	Once per 3 weeks	+32.4
	Once per 4 weeks	+30.0
	Once per 2 weeks	0
Side effects	Sedation	+200.6
	Sexual side effects (prolactin)	+107.2
	Weight gain of more than 7% of body mass	+102.6
	Extrapyramidal side effect	+79.1
	Metabolic side effects (diabetes, hypercholesterolaemia, dyslipidaemia)	+42.3
	Tardive dyskinesia	0
Pain at injection site	Absent	+158.8
	Mild	+158.7
	Moderate	+116.3
	Severe	0
Risk of embarrassment and potential harm to therapeutic relationship	Low risk	+118.6
	High risk	0
Routes of administration	Buttock muscle (gluteal)	+49.0
	Deltoid muscle	+45.4
	Ventro gluteal	0

Utility scores were also analysed by patient type (Table 3); this analysis revealed that side effects, pain at administration site, embarrassment and site of administration (gluteal versus deltoid) were important factors for all patients, regardless of patient type. There is a slight preference for injecting in the buttock, except for patients who have experienced two to five episodes then the deltoid muscle is slightly preferred.

**Table 3:** Utility scores split by patient type

**Attribute**	**Level**	**Utility scores**		
		**Patients who had their first episode**	**Patients who had 2 to 5 episodes**	**Patients who had 6 or more episodes**
Side effects	Sedation	+241.8	+235.4	+219.5
	Sexual side effects (prolactin)	+111.7	+156.4	+124.1
	Weight gain of more than 7% of body mass	+93.7	+136.1	+105.9
	Extrapyramidal side effect	+82.8	+123.4	+81.1
	Metabolic side effects (diabetes, hypercholesterolaemia, dyslipidaemia)	+70.0	+62.1	+27.4
	Tardive dyskinesia	0	0	0
Pain at injection site	Absent	+173.8	+169.1	+194.3
	Mild	+160.3	+163.1	+185.8
	Moderate	+130.2	+107.7	+145.7
	Severe	0	0	0
Risk of embarrassment and potential harm to therapeutic relationship	Low risk	+139.7	+120.0	+121.4
	High risk	0	0	0
Routes of administration	Buttock muscle (gluteal)	+67.0	+32.0	+74.4
	Deltoid muscle	+54.2	+29.3	+54.1
	Ventro gluteal	0	0	0

Analysis of results indicate that the preference for LAI antipsychotics were stronger for more chronic patients; 40% of the participating HCPs preferred LAI antipsychotics to oral treatment for patients experiencing a first episode of schizophrenia, 90% of respondents preferred LAI antipsychotics to oral medication for chronic patients with two to five episodes, and 96% preferred LAI antipsychotics to oral medication for patients with ≥6 episodes.

### Relative importance for LAI characteristics: analysis of attitudes and opinions

In this survey, attitudes towards LAI antipsychotics compared with orals were generally positive (Table 4). In particular, statements gaining the most agreement were that LAI antipsychotics allow intervention to address non-adherence (83% agreed), adherence advantages of LAI antipsychotics ensure a lower rate of relapse (87% agreed), and that LAI antipsychotics provide the best way to manage non-adherence due to poor insight (80% agreed). Sixty-one per cent of respondents agreed that deltoid administration may be a more respectful way of administering antipsychotics than gluteal administration. This statement was most strongly supported by HCPs in the UK (Table 4).

**Table 4:** Attitudes towards choice of antipsychotics for patients with schizophrenia – mean scores

**Means statements, oral versus LAI**	**All**	**The Netherlands**	**Belgium**	**Germany**	**Italy**	**UK**	**France**	**Nordic countries**
	**N = 891**	**n = 109**	**n = 97**	**n = 182**	**n = 187**	**n = 83**	**n = 132**	**n = 101**
1. If a patient is on a LAI antipsychotic and did not appear at the administration appointment as prescribed I can act upon it	5.4	5.6	5.1	5.6	5.8	5.6	4.6	5.7
2. If a patient is on oral antipsychotics, it is impossible to ascertain whether the patient has been taking an antipsychotic or not	4.3	4.4	4.9	3.3	4.7	4.1	4.5	4.2
3. Because of adherence advantages of LAIs, a lower rate of relapse can be ensured	5.5	5.4	5.5	5.7	5.6	5.2	5.5	5.2
4. The best way of managing non-adherence with antipsychotics (due to poor insight) is with LAIs	5.2	5.1	5.2	5.4	5.4	5.2	4.8	5.0
5. Administration of a LAI antipsychotic in the deltoid muscle as opposed to the buttocks is a respectful way of administering antipsychotics	4.6	4.7	4.6	4.4	4.4	5.2	4.7	5.0
6. The ability to administer a LAI antipsychotic in the deltoid muscle instead of the gluteal muscle will lead to an increase in the use of LAI antipsychotic medication	4.2	4.1	4.3	4.0	4.1	4.4	4.2	4.7
7. Current oral antipsychotics can get many patients well, but LAI atypical antipsychotics will keep the patients well	4.6	4.7	5.0	3.8	5.0	4.8	5.0	4.5

Respondents (Base: total n = 891) were asked to indicate, on a scale ranging from one to seven, whether they agreed or disagreed (1 = strongly disagree, 7 = strongly agree) with statements comparing long-acting medication with oral medication (question numbers 1–4 and 7) and statements comparing deltoid administration with oral administration (question numbers 5 and 6).

**Figure 2:**
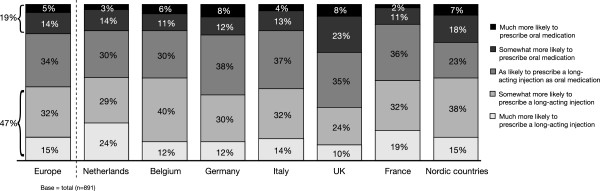
If a long-acting antipsychotic injection was available in the deltoid muscle, how would that influence your choice to prescribe a long-acting injection compared to oral medication?

**Figure 3:**
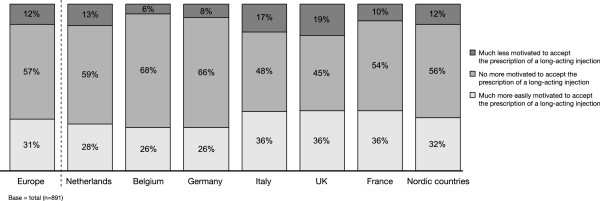
If a long-acting antipsychotic could be injected in the deltoid muscle, then, compared to oral medication, my patients would be.

**Figure 4:**
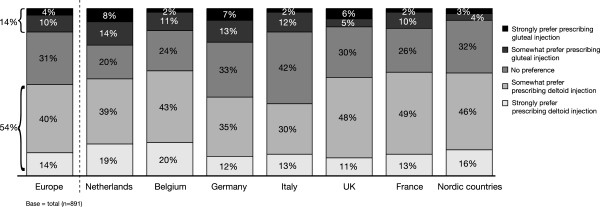
If a long-acting injection of antipsychotics was available in the deltoid muscle as well as in the buttocks (gluteal), how would this influence your prescription of long-acting antipsychotics?

**Figure 5:**
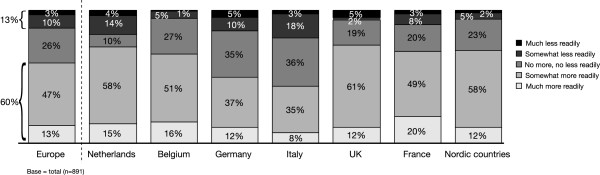
Compared to an injection in the buttock muscle, my patients would accept an injection in the deltoid muscle.

Evaluation of responses to statements regarding oral versus LAI medications showed that 47% of respondents would be more likely to choose an LAI antipsychotic than an oral formulation, if the choice of a deltoid administration site as well as the gluteal administration site were available (Figure 2). This response varied slightly according to country, with The Netherlands and the Nordic countries showing the most positive response (53%), and the UK showing the least positive response (34%). Overall, almost one third of respondents thought that their patients would be more easily motivated to accept LAI antipsychotics in preference to oral antipsychotics if the medication could be administered in the deltoid muscle (Figure 3). Given the choice of a deltoid or a gluteal administration site, 54% of respondents preferred deltoid to gluteal administration, compared with 14% who would prefer the gluteal option (Figure 4). Furthermore, 60% of respondents believed their patients would accept deltoid administration of LAI antipsychotic in preference to gluteal administration (Figure 5). This varied somewhat by country, with 73% of those in The Netherlands and UK believing that their patients would prefer deltoid, compared with 43% in Italy.

Sixty-seven per cent of respondents agreed that LAI antipsychotic administration in the deltoid muscle instead of the gluteal muscle decreases the social embarrassment involved. Overall, respondents considered that having the choice between gluteal and deltoid LAI antipsychotic administration sites was important (Table 5); 86% of respondents considered that having a choice between intramuscular (IM) administration in the deltoid or gluteal muscles was an advantage over not having this choice (where not having this choice is no IM administration into the deltoid muscle).

**Table 5:** Choices regarding LAI administration

**Means statements, oral versus LAI**	**All**	**The Netherlands**	**Belgium**	**Germany**	**Italy**	**UK**	**France**	**Nordic countries**
	**N = 891**	**n = 109**	**n = 97**	**n = 182**	**n = 187**	**n = 83**	**n = 132**	**n = 101**
1. Compared to administration in the gluteal muscle, administration in the deltoid muscle will help safeguard your therapeutic relationship with the patient by showing more respect for his or her dignity	4.4	4.6	4.3	4.1	4.2	4.9	4.4	4.8
2. IM administration in the deltoid muscle instead of the gluteal muscle decreases social embarrassment during administration	4.8	5.3	4.9	4.4	4.7	5.3	4.4	5.1
3. Administration in the gluteal muscle might incite some patients to make sexual connotations	3.9	4.3	3.3	3.6	4.1	4.1	4.0	4.2
4. IM administration in the deltoid muscle instead of the gluteal muscle is safer for the administrator as the patient is less likely to hit out during injection	3.2	3.2	3.1	2.7	3.5	3.3	3.1	3.8
5. Administration in the gluteal muscle might increase paranoia in some patients because they cannot see what is going on behind their back	4.0	4.3	4.1	3.6	4.1	3.9	4.1	4.3
6. Having a choice between IM administration in the deltoid or gluteal muscle is an advantage over not having this choice	5.6	6.0	5.4	5.1	5.7	5.8	5.6	5.9
7. Administration in the gluteal muscle is safer compared with administration in the deltoid muscle because nurses only have experience with administration in the gluteal muscle	3.4	3.8	3.1	3.6	3.0	4.0	3.4	3.5

Respondents (Base: total n = 891) were asked to indicate, on a scale ranging from one to seven, whether they agreed or disagreed (1 = strongly disagree, 7 = strongly agree) with statements comparing long-acting deltoid administration with gluteal administration (question numbers 1–7).

Overall, respondents did not agree with statements regarding safety of one method of administration over the other (Table 5). The statements that respondents disagreed with were ‘administration in the deltoid muscle instead of the gluteal muscle was safer for the administrator as the patient was less likely to hit out during injection’ and ‘administration in the gluteal muscle is safer compared with administration in the deltoid muscle because nurses only have experience with administration in the gluteal muscle’.

## Discussion

The objectives of this survey were to understand factors driving LAI use as well as attitudes and preferences for different administration sites, both in comparison with oral medication and with other LAI medication. Conjoint analysis was employed to examine trade-offs between specific antipsychotic characteristics including formulation, frequency of administration and route of administration to understand attitudes and preferences for different administration sites. Results of the survey provide insight to the attitudes of a large number of HCPs across Europe on the availability and use of LAI versus oral medication for the treatment of patients with schizophrenia. However, it is important to note that the HCPs surveyed did not evaluate antipsychotic treatment options directly. Rather, the conjoint analysis method employed calculates utilities based on respondents’ answers to specific trade-off questions. These utilities are then used to predict which option most closely suits each HCP’s individual preferences.

Conjoint analysis highlighted that side effects, pain at administration site, embarrassment and site of administration were all considered to be important factors by this group of HCPs when choosing between antipsychotic treatments, for both first episode and chronic schizophrenia patients. The majority of HCPs surveyed (96%) preferred LAI medications to oral treatment for patients with chronic schizophrenia whereas 40% preferred this type of medication for first episode patients. Further analysis indicates that there is a slight preference for injecting in the buttock, except for patients who have experienced two to five episodes, then the deltoid muscle is slightly preferred. Preference for oral formulation over LAI in first-episode patients is aligned with previously reported findings that, despite the adherence benefits of LAI antipsychotics, there is resistance amongst psychiatrists to use these medications for first-line treatment [31,32].

Attitudes towards LAI antipsychotics compared with oral medications were generally positive; most HCPs surveyed agreed that LAI antipsychotics allowed intervention to address non-adherence (83% agreed) and provided the best way to manage non-adherence due to poor insight (87% agreed). Non-adherence to antipsychotic medication is common in patients with schizophrenia, with at least one third of patients having problems with adherence in any given year and the majority of patients experiencing difficulties with medication adherence at some time during the course of their illness [41]. Reduced adherence is associated with increased risk of relapse [6], increased hospitalization [6] and a higher economic burden [14-16]. Intramuscular LAI antipsychotics can improve medication adherence compared with daily oral medication [18,32] due to their inherent sustained delivery of medication as well as the regular treatment monitoring by healthcare professionals. There is growing evidence that patients who remain on antipsychotic treatment experience additional benefits beyond symptomatic control, such as improvements in health-related quality of life [42]. This highlights the importance of educating patients and relatives on the consequences of partial and non-adherence and raising awareness of the treatment options available to overcome poor adherence to medication in psychosis. Furthermore, it is important to ensure HCPs receive up-to-date information on LAI and depot medication so as to promote a more positive attitude [43,44].

Despite their potential clinical benefits, a systematic review of attitudes of staff and patients noted that LAI antipsychotics are often only used as a last resort for the most stigmatized individuals [37]. Furthermore, a cross-sectional survey of patients, relatives and psychiatrists revealed that only 21% of patients without previous experience of injectable antipsychotics are informed of the option and only 9% are recommended to switch to an LAI antipsychotic by their psychiatrists [32]. In the survey reported here, HCPs considered side effects, pain associated with injection, embarrassment and site of administration were potential barriers to the use of LAI antipsychotics in both early stage and chronic patients with schizophrenia. No or mild administration site pain, minimal risk of embarrassment/damage to the therapeutic relationship and some sedation but no other side effects were ideal features of LAI antipsychotics identified by HCPs in the survey.

This survey of physicians and nurses from around Europe also revealed that having the choice of a deltoid as well as a gluteal administration site is perceived as beneficial over not having the choice of a deltoid administration. Respondents believed that the deltoid site may improve acceptance of LAI antipsychotics and be preferred by their patients. Two thirds of respondents agreed that deltoid administration may reduce social embarrassment associated with LAI antipsychotics. Most respondents (61%) said they believed that administration of LAI antipsychotics into the deltoid muscle as opposed to the gluteal muscle might be considered more respectful to the patient. A recent 25-week study, conducted in Europe and the USA, noted that approximately half of patients preferred deltoid to gluteal muscle injections with the most common reasons for this preference being that it was easier, less embarrassing, faster and more convenient than injection in the gluteal muscle [39]. The current survey suggests that the relevance of a deltoid administration site for patient preference and for the embarrassment associated with gluteal administration may vary by country and culture.

It is remarkable that the perception of the deltoid administration seemed to improve as the survey progressed: in the first conjoint analysis it was considered as possessing a negative utility, while in the reactions to the statements distilled from the literature it was received quite positively. Potentially this reflects a relative unfamiliarity with this mode of administration in practice at the time of the survey.

The availability of LAI antipsychotic deltoid administration, such as for RLAI and paliperidone palmitate, increases choice in LAI antipsychotic administration, allowing patients and HCPs the opportunity to select an administration site which may be perceived as more respectful and less socially embarrassing. The efficacy of RLAI has been demonstrated in a number of studies [21,45-47] and the deltoid and gluteal injections of RLAI are interchangeable in terms of drug exposure [27]. Maintenance doses of paliperidone palmitate are administered once-monthly, in either the deltoid or gluteal muscle. The efficacy of paliperidone palmitate for adult patients with schizophrenia has been demonstrated in several studies ranging from 9 to 52 weeks [48-51].

## Conclusions

The purpose of this study was to understand factors driving LAI antipsychotic use as well as attitudes and preferences for different administration sites, both in comparison with oral medication and with other LAI medication as previous studies have suggested that attitudes amongst HCPs may influence medication choices offered to patients [36]. The results of this survey highlight that the majority of HCPs would consider LAI antipsychotics in preference to oral medication for patients with chronic schizophrenia, whilst 40% of those surveyed would select this type of medication for first-episode patients. Attitudes towards LAI antipsychotics compared with orals were generally positive; the ability to identify and address non-adherence were considered key features of LAI antipsychotic compared with oral medication. Dominant elements in antipsychotic choice were low sedation but no tardive dyskinesia, no or mild pain at injection and low risk of embarrassment or impact upon therapeutic alliance. The choice of a deltoid as well as a gluteal LAI antipsychotic administration site was perceived as beneficial over not having the choice of a deltoid administration. The availability of a deltoid administration therefore offers additional opportunity in the mode of LAI antipsychotic administration.

### Limitations

Our results must be considered in view of the limitations of the study. Conjoint analysis does not directly evaluate antipsychotic treatment options but calculates utilities to predict which treatment options most closely suits HCPs preferences. It also assumes that utilities for individual treatment characteristics are additive and does not permit exploration of interaction effects. In addition, as with all questionnaires, the description of the attributes may influence respondents’ judgment. However, conjoint analysis can also help overcome the known difficulties in communicating complex risk information. One of the main advantages of choice-based conjoint analysis is that it is interactive, allowing the investigator to evaluate a large number of attributes without information overload or respondent fatigue. This is particularly important as often the majority of complex medical decisions involve multiple trade-offs. In addition, choice-based conjoint analysis constructs utilities based on trade-offs between specific treatment characteristics. This minimizes the biases associated with the context in which choices are presented. Additionally, treatment characteristics can be presented in random order, thus eliminating potential ordering effects. Furthermore, by asking respondents to consider specific treatment advantages and disadvantages, it makes trade-offs between competing options explicit which has been shown to improve the quality of decision-making.

The nature of the recruitment process does create a certain selection bias, as only contacted physicians who were willing to complete the online questions were included. Even though they were randomly selected from a much larger list, a purely random sample could have been more appropriate. This was not performed to ensure adequate numbers of respondents. However, the majority of participants had extensive clinical experience (≥8 years), treating patients in a variety of settings including specialist psychiatric hospitals, general hospitals and medical psychological centres, and represented countries across Europe. While interpreting the results of such a discrete choice experiment, the effect of prior knowledge on the decision problem should be taken into account. In this survey, clinical experience with LAI antipsychotics may influence physician and nurse attitudes regarding this type of administration routes compared with those respondents without extensive experience of long-acting medication. Furthermore, it is important to acknowledge that the questions covered in this choice-based survey required participants to choose between bundles of predefined attributes; the limitation of this design is that such stated opinions may not reflect actual opinion or predict actual behaviour.

The current survey involved multidisciplinary HCPs (physicians/specialists: 78%; nurses: 22%). Attitudes and preferences for physicians versus nurses or psychiatrists versus non-psychiatrists were not analysed here. Although such analysis could provide insight into differences between HCPs, this was beyond the scope of this study. Furthermore, the inclusion of both psychiatrists and nurses reflects the situation in clinical practice with multidisciplinary teams involved in developing treatment plans.

## Competing interests

AS, PG and GM are all employees of Janssen.

## Authors’ contributions

PG was involved in the set-up and analysis of the survey. Both AS and GM were instrumental in the content and development of this manuscript. All authors read and approved the final manuscript.

## Pre-publication history

The pre-publication history for this paper can be accessed here:

http://www.biomedcentral.com/1471-244X/13/58/prepub
